# Discriminative performance of the clock drawing test for screening cognitive function in hypertensive patients aged ≥ 50 years: a cross-sectional study

**DOI:** 10.1186/s12875-026-03334-1

**Published:** 2026-04-18

**Authors:** Ozturk Gurer Tutu, Zeynep Ziroglu, Veli Bilen, Cansu Tutu, Nuriye Yilmaz

**Affiliations:** 1 Osmaniye Research and Training Hospital, Family Medicine Clinic, Osmaniye, 80000 Türkiye; 2 Ankara Research and Training Hospital, Neurology Clinic, Ankara, 06230 Türkiye; 3 Department of Family Medicine, Hatay Mustafa Kemal University, Hatay, Türkiye; 4 Neurology Clinic, Osmaniye Research and Training Hospital, Osmaniye, 80000 Türkiye; 5 Gerontology Department, Osmaniye Research and Training Hospital, Osmaniye, 80000 Türkiye

**Keywords:** Primary care, Hypertension, Cognitive impairment, Clock Drawing Test, Cognitive screening

## Abstract

**Background:**

Hypertension is associated with an increased risk of cognitive impairment, yet brief and practical screening tools suitable for routine clinical settings remain needed. This study evaluated diagnostic accuracy of the Clock Drawing Test (CDT) for identifying screen-positive cognitive impairment in hypertensive adults aged ≥ 50 years.

**Methods:**

We conducted a cross-sectional study among 365 patients with essential hypertension attending neurology and family medicine outpatient clinics of a secondary-care hospital in Turkey. CDT was administered by two trained family physicians using a standardized instruction and scored using two methods: CDT-1 (0–4 points) and CDT-2 (dichotomous normal/abnormal; scored as 1/0). The Montreal Cognitive Assessment (MoCA) and Mini-Mental State Examination (MMSE) were administered by a gerontologist blinded to CDT results; CDT, MoCA, and MMSE were scored independently by neurologists blinded to each other’s scores and to other cognitive test results. Screen-positive cognitive impairment was operationally defined as MoCA ≤ 21 (primary reference) and low global cognitive performance as MMSE < 24 (secondary reference). Diagnostic accuracy was evaluated using receiver operating characteristic (ROC) analyses, including the area under the ROC curve (AUC), sensitivity, and specificity at clinically feasible CDT cut-offs.

**Results:**

The mean age was 63.7 ± 7.9 years; 53.5% were male. The prevalence of screen-positive cognitive impairment was 49.9% by MoCA ≤ 21 (182/365) and 33.2% by MMSE < 24 (121/365). CDT-1 demonstrated good discrimination against MoCA ≤ 21 (AUC 0.830; 95% confidence interval [CI] 0.787–0.873) and MMSE < 24 (AUC 0.834; 95% CI 0.787–0.881). For MoCA ≤ 21, sensitivity/specificity were 62.1%/91.3% at CDT-1 ≤ 2 and 83.0%/65.6% at CDT-1 ≤ 3. For MMSE < 24, sensitivity/specificity were 74.4%/84.0% at CDT-1 ≤ 2 and 88.4%/56.1% at CDT-1 ≤ 3.

**Conclusions:**

In hypertensive adults aged ≥ 50 years, CDT-1 showed good overall discrimination and a clear sensitivity–specificity trade-off depending on the cut-off, supporting its use as a rapid pre-screening/triage tool to identify individuals who may warrant more comprehensive cognitive assessment.

**Supplementary Information:**

The online version contains supplementary material available at 10.1186/s12875-026-03334-1.

## Introduction

Hypertension is a major risk factor for cognitive impairment and brain dysfunction and is increasingly recognised as a key contributor to vascular and mixed dementias. Since the 1960s, accumulating evidence has demonstrated that hypertension is associated with deficits in abstract reasoning, memory, attention, and processing speed—cognitive domains that constitute early features of vascular cognitive impairment [1]. Importantly, these alterations often develop insidiously and may remain clinically unrecognised for years, underscoring the critical importance of early cognitive assessment in the prevention, timely detection, and management of later-life neurological disorders, including dementia.

Cognitive impairment is highly prevalent among hypertensive individuals aged over 50 years, particularly in older populations, although reported prevalence rates vary substantially across studies and populations [2, 3]. In a large clinical study, cognitive impairment was identified in 24% and 30% of hypertensive patients aged 50–89 years using the Mini-Mental State Examination (MMSE) and the Mini-Cog, respectively [4]. Consistent with these findings, a systematic review estimated the pooled prevalence of mild cognitive impairment (MCI) among hypertensive patients to be approximately 30% [5]. Notably, even higher prevalence rates have been reported in advanced age groups; for example, cognitive impairment was observed in 61.4% of hypertensive individuals aged over 65 years in one cohort, highlighting the substantial cognitive burden associated with hypertension in later life [6].

Hypertension represents the most important modifiable risk factor for brain damage and typically precedes clinically manifest cognitive impairment by more than a decade [7, 8]. It preferentially affects executive function and semantic memory through subcortical vascular injury [8], while in individuals with Alzheimer’s disease, coexisting vascular pathology is associated with earlier symptom onset and accelerated disease progression [9]. Beyond macroscopic vascular injury, hypertension-related vascular damage contributes to both cortical and subcortical cognitive impairment by disrupting the blood–brain barrier and impairing amyloid-β clearance [10–12], thereby placing hypertensive individuals at increased risk for both amnestic and non-amnestic cognitive decline [13].

Clinically silent cerebrovascular lesions—including white matter hyperintensities (WMHs), lacunes, cerebral microbleeds, and enlarged perivascular spaces—are common sequelae of hypertension and are strongly linked to cognitive decline [14]. WMHs, in particular, are a hallmark of slowly progressive vascular cognitive impairment and approximately double the risk of dementia. Their occurrence is closely associated with hypertension, especially elevated diastolic blood pressure (DBP), independent of age [14]. Wartolowska et al. reported that increases in DBP before the age of 50 were more predictive of WMH burden than systolic blood pressure [15]. Furthermore, a large meta-analysis demonstrated that both systolic and diastolic blood pressure are independently associated with WMH progression, regardless of mean arterial pressure [16].

Taken together, the high prevalence of cognitive impairment in hypertensive populations, its prolonged subclinical phase, and the central role of vascular pathology highlight the need for brief, reliable, and easily deployable cognitive screening tools in routine clinical settings. In this context, screening instruments that are sensitive to executive and visuospatial dysfunction—domains particularly vulnerable to vascular injury—are of particular clinical relevance.

The Clock Drawing Test (CDT) fulfills this need as a simple and rapidly administered instrument that assesses multiple cognitive domains commonly affected in dementia, including visuospatial ability, executive function, memory, and language, and has demonstrated utility in detecting early-stage dementia [17–19].

During CDT performance, distinct error patterns may provide insight into specific cognitive deficits: errors in numerical placement may reflect impairments in memory or orientation, whereas inaccurate placement of clock hands is often associated with deficits in executive function and planning. The task also requires intact hand–eye coordination and fine motor skills [17, 18]. Numerous CDT scoring methods have been described and are broadly categorized as quantitative, semi-quantitative, or qualitative, each with specific strengths and limitations. Quantitative methods tend to better differentiate dementia from normal cognition, whereas qualitative approaches may be more informative for distinguishing dementia subtypes [20]. Quantitative and semi-quantitative scoring systems further offer the advantage of rapid scoring, which is particularly relevant in time-constrained clinical settings [21, 22].

Against this background, the present study aimed to evaluate the diagnostic accuracy of the CDT as a rapid screening tool for cognitive impairment in hypertensive adults aged ≥ 50 years. The Montreal Cognitive Assessment (MoCA) ≤ 21 was used as the primary reference screening threshold, with MMSE < 24 serving as a secondary reference threshold for low global cognitive performance. Clarifying the screening performance of the CDT in this population may facilitate earlier identification of screen-positive cognitive impairment in family medicine settings and support timely referral for further assessment and intervention.

## Materials and methods

### Participants

The present study constitutes a cross-sectional investigation, encompassing patients over the age of 50 years with essential hypertension who attended the neurology and family medicine outpatient clinics of a secondary hospital in Turkey. Throughout the study, the 1975 Declaration of Helsinki criteria were adhered to. The study was conducted between February 2025 and June 2025. The study included 365 volunteers aged over 50 years with essential hypertension who had at least primary school education (≥ 5 years of formal schooling). Accordingly, illiterate individuals were not included in the study sample. Individuals were excluded if they had a history of stroke, dementia, Parkinson’s disease, psychotic disorders, depressive disorders, or substance/alcohol dependence. We also excluded those with active malignancy, those undergoing chemotherapy, those with active infection, pregnant women, individuals who lacked capacity to provide informed consent, those with acquired immunodeficiency disease, and those using immunosuppressive drugs.

We recorded the number of individuals who declined participation. For non-participants, only age and sex were available from administrative records; no further sociodemographic or cognitive test data (including education and CDT/MMSE/MoCA scores) could be collected because they did not provide consent for study assessment.

### Procedure

Eligible patients attending the neurology and family medicine outpatient clinics during routine visits were recruited consecutively. After obtaining written informed consent, vital signs (including blood pressure) were measured by trained nurses as part of standard care. Nurses had no role in the administration or scoring of any cognitive assessments.

The CDT was administered by trained family physicians (*n* = 2) in a quiet room using a standardized instruction (“Please draw an analogue clock, put in all the numbers, and set the hands to 11:10”) on a blank A4 sheet with a pencil.

Global cognitive screening tests (MoCA and MMSE) were administered by a gerontologist who was not involved in CDT administration and was blinded to CDT results. CDT drawings were scored by a neurologist, while MoCA and MMSE were scored by a different neurologist. The two neurologist raters scored independently and were blinded to each other’s scores and to the other cognitive test results.

### Data collection tools

#### Sociodemographic and clinical data

A demographic data form was completed for participants, and the duration of hypertension, medications used, and average systolic/diastolic blood pressure were recorded. The measurement of blood pressure was conducted utilising a standardised OMRON M10 device, with the results being defined in accordance with the guidelines established by the American Heart Association (AHA) [23]. The participants were distributed across four age categories (50–59, 60–69, 70–79, and ≥ 80 years). Educational level was categorized into four groups: Primary school, Middle school, High school, and University. The documentation of concomitant illnesses and medications was meticulously recorded for each patient.

##### Hospital anxiety and depression scale (HADS)

The HADS, developed by Zigmond and Snaith in 1983, is designed to rapidly screen for anxiety and depression in patients with physical illnesses rather than to establish a diagnosis [24]. Higher scores indicate greater anxiety and depression, with a total score ranging from 0 to 21; scores of 0–7 are considered normal. The Turkish validity and reliability study was conducted by Aydemir et al. in 1997 [25].

##### Mini mental state examination

Mini Mental State Examination (MMSE), developed by Folstein and McHugh in 1975, is widely used as a brief measure of global cognitive status [26]. The total score ranges from 0 to 30. In this study, we used MMSE < 24 as a secondary reference screening threshold indicating low global cognitive performance, consistent with the Turkish validation study by Güngen et al. (2002) [27].

##### Montreal cognitive assessment (MoCA)

MoCA is a screening instrument developed to detect milder cognitive deficits and is generally more sensitive than MMSE for early cognitive changes [28]. The total score ranges from 0 to 30. In this study, we used MoCA ≤ 21 as the primary reference screening threshold to operationally define “screen-detected cognitive impairment,” based on the Turkish validation by Selekler et al. (2010) [29].

##### Clock drawing test (CDT)

The CDT is a brief neuropsychological screening tool that evaluates multiple cognitive domains, including visuospatial skills, motor planning, attention, memory, and executive functions, and it is considered a rapid and efficient method for cognitive screening in clinical practice [17]. The CDT was administered in a standardized manner: participants were instructed to draw an analogue clock, place the numbers correctly, and set the hands to 11:10.

In the literature, several CDT scoring methods have been described (e.g., 10-point, 5-point, 4-point, and dichotomous 1–0 approaches), each offering different trade-offs between scoring granularity and ease of implementation [18, 22, 30]. In this study, we applied two scoring approaches. CDT-1 used the Turkish validated 4-point scoring system described by Cangöz et al. (2006), yielding a total score from 0 to 4, with higher scores indicating better performance [30]. CDT-2 used a simplified dichotomous (1–0) scoring method (normal/abnormal) to reflect a pragmatic approach that can be implemented rapidly in busy primary care settings. In CDT-2, scoring was intentionally stringent: a drawing was rated ‘normal’ (score = 1) only if all essential clock elements were correctly placed; any error (even minor) resulted in ‘abnormal’ (score = 0) [18, 22, 30].

We selected these two versions to compare a locally validated semi-quantitative method (CDT-1) with a simplified binary method (CDT-2), thereby assessing potential trade-offs between diagnostic accuracy and operational simplicity when using CDT as a rapid screening tool in routine care [18, 22, 30].

Because participants did not undergo a clinical diagnostic assessment for mild cognitive impairment or neurocognitive disorder, the outcome in this study was defined operationally. We defined screen-detected cognitive impairment as a MoCA score ≤ 21, based on the Turkish validation study. A MMSE score < 24 was analyzed as a secondary reference threshold reflecting lower global cognitive performance rather than a clinical diagnosis of early cognitive impairment. These reference thresholds originate from different case definitions (MMSE 23/24 largely from dementia–control comparisons, whereas MoCA cut-offs are typically derived to detect milder impairment such as MCI versus normal cognition); therefore, higher screen-positive prevalence using MoCA than MMSE is expected.

### Statistics

Statistical analyses were performed using IBM SPSS version 21.0. Descriptive statistics were presented as frequency, mean, standard deviation, and percentage. Data normality was assessed using the Shapiro–Wilk test. The Mann–Whitney U test was used for non-normally distributed continuous variables, and categorical variables were analyzed using the chi-square or Fisher’s exact test as appropriate. Statistical significance was set at *p* < 0.05 with a 95% confidence interval. Sample size was calculated using G*Power to achieve 80% power (1 − β = 0.80) and a 95% confidence interval with a 5% margin of error. Based on a reported maximum prevalence of cognitive impairment of 30% among hypertensive individuals aged ≥ 50 years [4–6], the minimum required sample size was 323 and was increased to 365 to account for potential data loss. Associations between blood pressure control categories and cognitive impairment (defined by MMSE, MoCA, and CDT cut-offs) were explored using categorical comparisons (chi-square/Fisher’s exact test). Diagnostic accuracy was assessed using sensitivity, specificity, and receiver operating characteristic (ROC) curve analyses.

## Results

### Sociodemographic and clinical characteristics of the participants

The study included 365 participants with a mean age of 63.7 ± 7.9 years (range: 50–93); 53.5% were male and 46.5% female. Of the individuals approached, 77 declined participation. Compared with participants, non-participants did not differ significantly in age (63.7 ± 7.9 vs. 64.6 ± 7.6 years; *p* = 0.361) or sex distribution (female: 46.5% vs. 45.4%; *p* = 0.858) (Supplementary Table 1). Male participants were older and had higher education levels than females (*p* < 0.05; *p* < 0.001). Participants were similarly distributed across hypertension duration categories (0–5, 5–10, 10–15, and > 15 years: 25.7%, 24.7%, 26.8%, and 22.7%, respectively). Mean systolic, diastolic, and mean arterial pressure (MAP) were 135.5 ± 17.5, 80.6 ± 11.7, and 98.9 ± 12.3 mmHg, respectively. SBP was controlled in 55.3% (*n* = 202) and DBP in 76.2% (*n* = 278) of participants (Table 1).

**Table 1:** Baseline characteristics (*n* = 365)

Variables	Total Sample *n* (%)	Female, *n* (%)	Male, *n* (%)	*p*
Sample	365	170 (46.5)	195 (53.5)	
Age (Years) (M ± SD)	63.7 ± 7.9	62,9 ± 7.7	64,3 ± 7.9	0.025*
Age (Category of year)				
50–59 60–69 70–79 80 & above	113 (31.0) 169 (46.3) 73 (20.0) 10 (2.7)	60 (35.3) 76 ( 44.7) 29 (17.1) 5 (2.9)	53(27.2) 93 (47.7) 44 (22.6) 5 (2.6)	0.312 ^‡^
Education Level				
Primary school Middle school High school University	224 (61.3) 36 (9.9) 54 (14.8) 51 (14.0)	127 (74.7) 8 (4.7) 19 (11.2) 16 (9.4)	97 (49.7) 28 (14.4) 35 (17.9) 35 (17.9)	< 0.001^†^
Marital Status				
Married Single	345 (94.0) 20 (6.0)	162 (95.3) 8 (4.7)	183 (93.8) 12 (6.2)	0.544^†^
Chronic Disease				
Diabetes Mellitus Dyslipidemia CAD	151 (41.0) 72 (20.0) 73 (20.0)	71 (41.8) 34 (20.0) 24 (14.1)	80 (41.0) 38 (19.5) 49 (25.1)	0.886^†^ 0.902^†^ 0.090^†^
Medication				
ACE inhibitör ARB Beta-blocker Alfa- blocker Diuretic CCB	145 (40.0) 150 (41.0) 124 (34.0) 15 (4.0) 174 (48.0) 141 (39.0)	59 (34.7) 84(49.4) 57 (33.5) 3 (1.8) 86 (50.6) 63 (37.1)	86 (44.1) 66(33.8) 67 (34.4) 12 (6.2) 88 (45.1) 78 (40.0)	0.067^†^ 0.003^†^ 0.867^†^ 0.035^†^ 0.297^†^ 0.565^†^
Duration of Hypertension (years)				
0–5 5–10 10–15 > 15	94 (25.7) 90 (24.7) 98 (26.8) 83 (2.7)	34 (20.0) 39 (22.9) 49 (28.8) 48 (28.2)	60 (30.8) 52 (26.7) 49 (25.1) 34 (17.4)	0.021^†^
Systolic BP (mmHg) (M ± SD) Normal Abnormal	135.5 ± 17.5 202 (55.3) 163 (44.7)	134.39 ± 16.1 93 (54.7) 77 (45.3)	137.69 ± 19.1 109 (55.9) 86 (44.1)	0.285* 0.819^†^
Diastolic BP (mmHg) (M ± SD) Normal Abnormal	80.6 ± 11.7 278 (76.2) 87 (23.8)	80.0 ± 11.0 135 (79.4) 35 (20.6)	82.2 ± 12.4 143 (73.3) 52 (26.7)	0.155* 0.174^†^
MAP (M ± SD)	98.9 ± 12.3	99.6 ± 11.5	98.3 ± 13.0	0.197*

*Mann-Whitney U Test/ † Chi-Square Test/ ‡ Fisher’s Exact Test; *MAP* Mean Arterial Pressure, *BP* Blood Pressure, *ACE* Angiotensin-Converting Enzyme, *ARB* Angiotensin Receptor Blocker, *CCB* Calcium Channel Blocker

### Neurocognitive test results of the participants

Mean HADS-Anxiety (HADS-A) and HADS-Depression (HADS-D) scores were 7.3 ± 4.9 and 6.3 ± 4.2, respectively. The prevalence of cognitive impairment based on MMSE, MoCA, CDT-1, and CDT-2 was 33.2%, 49.9%, 35.4%, and 58.6%, respectively. Male participants had higher mean MoCA and CDT scores, while MMSE, HADS-A, and HADS-D scores did not differ significantly between gender (Table 2).

**Table 2:** Distribution of cognitive test results

Test	Total Sample *n* (%)	Female *n* = 170 *n* (%)	Male *n* = 195 *n* (%)	*p*
MMSE (M ± SD)	24.8 ± 4.4			0.088
≥ 24 (Normal) < 24 (Abnormal)	244 (66.8) 121 (33.2)	106 (62.4) 64 (37.6)	138 (70.8) 57 (29.2)	
MoCA (M ± SD)	21.1 ± 4.3			**0.021**
> 21 (Normal) ≤ 21 (Anormal)	183 (50.1) 182 (49.9)	74 (43.5) 96 (56.5)	109 (55.9) 86 (44.1)	
Clock Drawing Test-1				**0.029** ^**a**^
0 1 2 3 4	4 (1.1) 55 (15.1) 70 (19.2) 85 (23.3) 151 (41.4)	3 (1.8) 30 (17.6) 35 (20.6) 41 (24.1) 61 (35.9)	1 (0.5) 25 (12.8) 35 (17.9) 44 (22.6) 90 (46.2)	
Clock Drawing Test-2				0.047
Normal Abnormal	151 (41.4) 214 (58.6)	61 (35.9) 109 (64.1)	90 (46.2) 105 (53.8)	
HADS-Depression (M ± SD)	6.3 ± 4.2			0.304
Normal Abnormal	208 (57.0) 157 (43.0)	90 (52.9) 80 (47.1)	108 (55.4) 87 (44.6)	
HADS-Anxiety (M ± SD)	7.3 ± 4.9			0.304
Normal Abnormal	194 (52.9) 171 (47.1)	85 (50.0) 85 (50.0)	108 (55.4) 87 (44.6)	

p-value: Bold values indicate statistically significant results (p<0,05). For categorical variables, the Chi-Square test was used. ^a^If any of the expected cell counts were less than 5, Fisher’s Exact Test result was used

### Diagnostic accuracy of the clock drawing test

ROC analyses showed that CDT-1 had good overall discrimination, with an AUC of 0.834 (95% CI: 0.787–0.881) when referenced against low MMSE performance (MMSE < 24) and 0.830 (95% CI: 0.787–0.873) when referenced against screen-detected cognitive impairment (MoCA ≤ 21). Because CDT-1 is integer-scored, we report diagnostic performance at the clinically feasible cut-offs of ≤ 2 and ≤ 3, demonstrating the expected sensitivity–specificity trade-off: for MMSE < 24, sensitivity increased from 74.4% at ≤ 2 to 88.4% at ≤ 3, while specificity decreased from 84.0% to 56.1%; similarly, for MoCA ≤ 21, sensitivity increased from 62.1% to 83.0% and specificity decreased from 91.3% to 65.6%. In comparison, CDT-2 yielded lower discrimination, with AUCs of 0.723 (95% CI: 0.670–0.776) against MMSE < 24 and 0.749 (95% CI: 0.698–0.801) against MoCA ≤ 21 (Table 3).

**Table 3:** Diagnostic performance of the clock drawing test

	Criterion Test	AUC (95% CI)	Cut-off Point (CDT)	Sensitivity (%)	Specificity (%)	PPV (%)	NPV (%)
CDT-1	MoCA ≤ 21	0.830 (0.787–0.873)	≤ 2 ≤ 3	62.1 83.0	91.3 65.6	87.6 70.6	70.8 79.5
	MMSE < 24	0.834 (0.787–0.881)	≤ 2 ≤ 3	74.4 88.4	84.0 56.1	69.8 50.0	86.9 90.7
CDT-2	MMSE < 24	0.723 (0.670–0.776)	Abnormal (score = 0)	88.4	56.1	50.0	90.7
	MoCA ≤ 21	0.749 (0.698–0.801)	Abnormal (score = 0)	83.0	65.6	70.6	79.5

*AUC* Area Under Curve, *CI *Confidence Interval, *PPV *Positive Predictive Value, *NPV *Negative Predictive Value

**Fig. 1:**
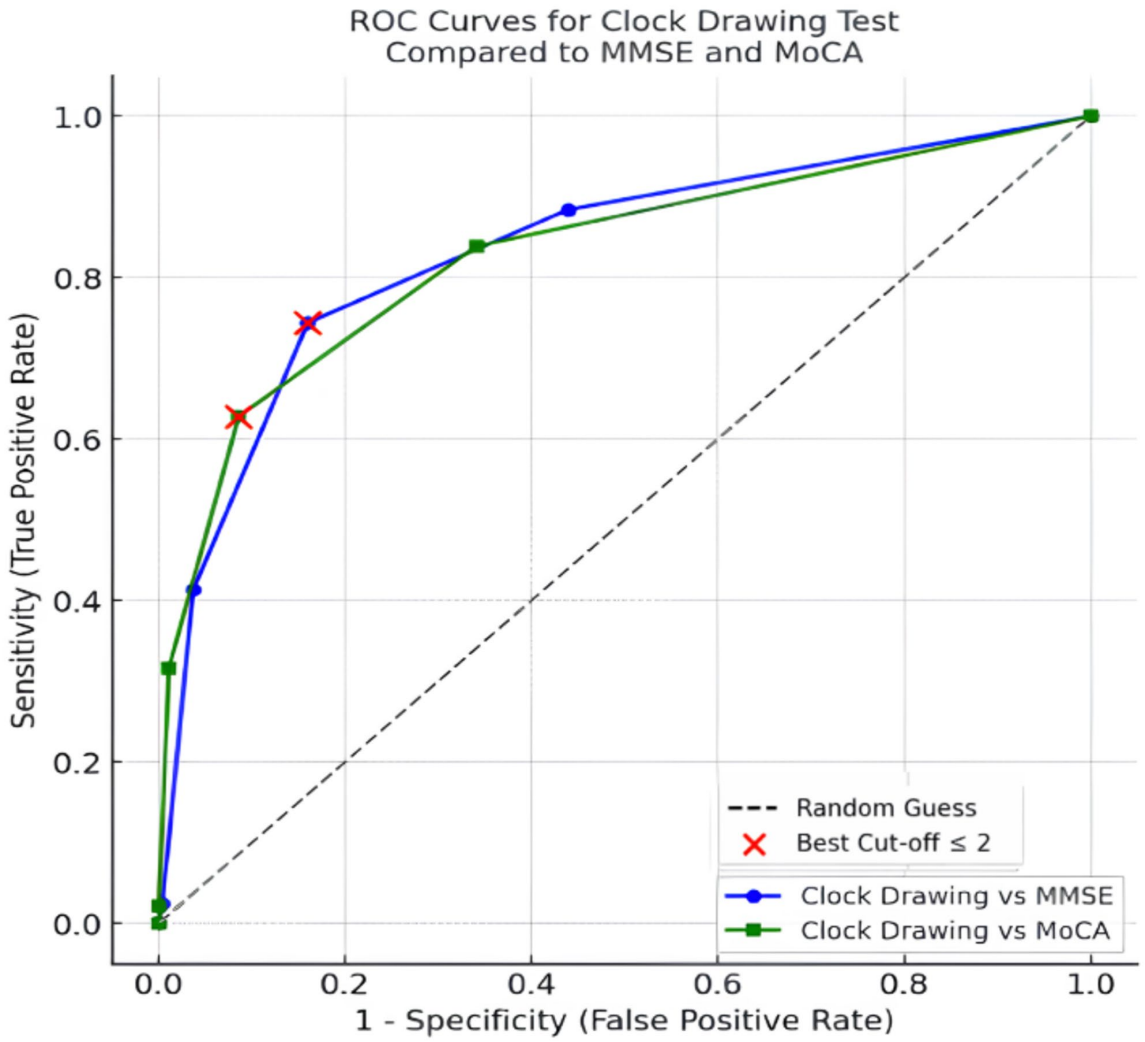
ROC curve coordinates for clock drawing test-1 for identifying screen-detected cognitive impairment (MoCA ≤ 21) and low global cognitive performance (MMSE < 24)

ROC curve analysis suggested an optimal non-integer threshold of approximately 2.5 for CDT-1 against both reference standards. Because CDT-1 is scored in whole numbers, this corresponds to a practical clinical cut-off of ≤ 2, at which sensitivity and specificity were 74.4% and 84.0%, respectively, when referenced against low MMSE performance (MMSE < 24), and 62.1% and 91.3%, respectively, when referenced against screen-detected cognitive impairment (MoCA ≤ 21) (Fig. 1). Consistent with the expected sensitivity–specificity trade-off, adopting a higher integer threshold (≤ 3) increased sensitivity while reducing specificity across both reference standards (Table 3).

### Correlations between cognitive test scores and affective measures

**Fig. 2:**
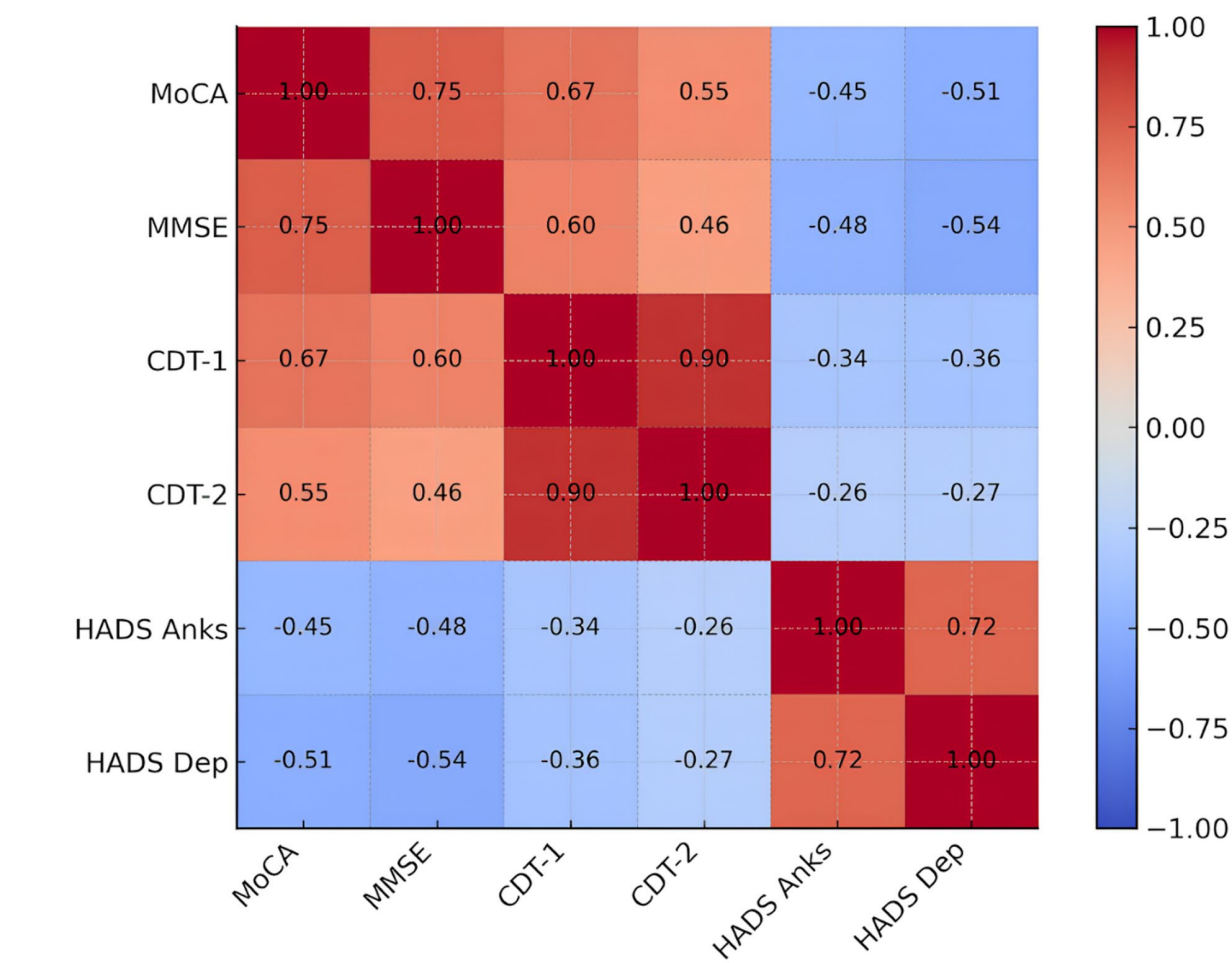
Spearman correlation matrix among the study variables **(**Lighter shades indicate stronger correlations. Positive and negative correlations are color-coded.)

In the analysis results, significant positive correlations were found among all cognitive test scores. The MoCA score showed a strong positive relationship with the MMSE (rₛ= 0.749; *p* < 0.001), CDT-1 (rₛ = 0.673; *p* < 0.001), and CDT-2 (rₛ = 0.553; *p* < 0.001). Similarly, the MMSE was significantly positively correlated with CDT-1 (rₛ = 0.604; *p* < 0.001) and CDT-2 (rₛ = 0.458; *p* < 0.001). HADS-A and HADS-D scores were strongly positively correlated (rₛ = 0.719; *p* < 0.001) and both showed significant inverse correlations with cognitive test scores, including the MoCA (anxiety: rₛ = − 0.448; depression: rₛ = − 0.505; *p* < 0.001) (Fig. 2).

## Discussion

In the present study, CDT-1 demonstrated high specificity with moderate sensitivity for identifying screen-positive cognitive impairment when referenced against predefined MMSE and MoCA thresholds in hypertensive adults aged ≥ 50 years in primary care. We acknowledge that sensitivity below 80% particularly at the ≤ 2 cut-off against MoCA should not be interpreted as uniformly “good” for a stand-alone screening test. Rather, our results indicate a clear trade-off: ≤2 prioritizes specificity (fewer false positives), whereas ≤ 3 increases sensitivity at the cost of lower specificity, supporting the CDT as a rapid pre-screening/triage tool to identify individuals who may warrant more comprehensive cognitive assessment. Accordingly, the preferred cut-off may depend on clinical priorities. A threshold of ≤ 3 may be considered when maximizing sensitivity is important, whereas ≤ 2 may be preferred in settings where confirmatory assessment is available and minimizing false positives is desirable. For example, when using MMSE < 24 as the reference, sensitivity increased from 74.4% (≤ 2) to 88.4% (≤ 3) while specificity decreased from 84.0% to 56.1%; similarly, against MoCA ≤ 21, sensitivity increased from 62.1% to 83.0% while specificity decreased from 91.3% to 65.6%.

Using the 1–0 and Turkish-validated 4-point CDT scoring systems described by Cangöz et al. [30]. CDT-1 demonstrated sensitivity/specificity values of 74.4%/84.0% against MMSE and 62.1%/91.3% against MoCA, with an AUC of approximately 0.83. These findings indicate that CDT-1 has high specificity for identifying screen-positive cognitive impairment based on MoCA ≤ 21 (and, secondarily, low MMSE performance defined as MMSE < 24), supporting its use as a rapid pre-screening tool rather than a stand-alone alternative to MoCA. In contrast, CDT-2 showed lower diagnostic accuracy (AUC: 0.723 with MMSE; 0.749 with MoCA). CDT-2 showed higher sensitivity but lower specificity than CDT-1 at the ≤ 2 threshold, reflecting a more liberal classification at the expense of false positives.On this basis, CDT-1 appears more suitable for rapid pre-screening or triage in hypertensive individuals, whereas CDT-2 may be useful as an initial triage tool in primary care settings where examiner time or training is limited, provided that results are interpreted alongside more detailed cognitive assessments. Consistent with prior studies, better CDT performance in hypertensive older adults has been associated with a lower risk of dementia [31]. A study reported that CDT is a more sensitive screening test than MMSE in hypertensive patients because it better demonstrates executive functions, and CDT impairment was observed in a proportion of hypertensive individuals with normal MMSE scores, suggesting that CDT may flag screen-positive cognitive deficits that are not captured by MMSE alone [7].

Beyond classification performance, the correlation pattern supports the construct validity of CDT in this setting. CDT-1 showed a clearer alignment with global cognitive measures (MMSE/MoCA), whereas the association of CDT scores with mood symptoms were weaker than that observed for MMSE/MoCA. This distinction is clinically relevant, as mood-related symptoms may depress performance on global cognitive screeners and inflate screen-positive rates if not appropriately contextualized. In hypertensive older adults, comparative studies similarly suggest that different screening tools vary in their sensitivity to domain-specific deficits, reinforcing the importance of interpreting CDT results alongside broader cognitive measures rather than as a stand-alone substitute [32].

In our dataset, sensitivity, specificity, PPV and NPV were identical when the CDT-1 cut-off was set at ≤ 3 and compared with CDT-2. This finding is best understood as a consequence of the CDT-2 scoring logic rather than a true redundancy between the tests. Because CDT-2 defines “normal” only in the presence of complete correctness (score = 1) and labels any deviation as “abnormal” (score = 0), it implements a highly stringent dichotomous classification, which can yield a 2 × 2 table that is operationally equivalent to a semi-quantitative threshold depending on the score distribution. This interpretation is consistent with prior evidence indicating that different CDT scoring methods may show broadly comparable overall discrimination in certain settings, with no single approach consistently superior across comparisons [33–35]. Nevertheless, dichotomous ratings reduce granularity and may be less informative than ordinal scales; for example, clinician-rated CDT using an ordinal (0–10) scale has been reported to be more predictive than binary impaired/intact classifications, highlighting potential information loss with strict dichotomization [34].Accordingly, the equivalence observed here should be interpreted as context-dependent and may not generalize to populations with different severity profiles or score distributions.

Sex-related differences in cognitive impairment have been reported in hypertensive populations, with some studies suggesting a higher risk among women [8, 36], while others found no association between sex and CDT performance [37]. Depression and anxiety have also been reported to be more prevalent among women [7]. In the present study, male participants performed better on the MoCA, CDT-1, and CDT-2, whereas anxiety and depression scores did not differ significantly by gender. Consistent with prior evidence, anxiety and depression were more strongly negatively associated with MMSE and MoCA scores, while their relationship with CDT performance was weaker [7, 38]. These findings suggest that mood disorders should be considered when interpreting cognitive screening results.

The observed proportions of screen-positive cognitive impairment should be interpreted as threshold-based screening outcomes rather than clinical diagnoses. Differences in screen-positive rates between MMSE and MoCA are expected, given their differing sensitivity to milder cognitive impairment, and may also be influenced by educational level and comorbid symptom burden. Accordingly, the prevalence estimates reported here reflect the burden of screen-positive cognitive impairment under predefined cut-offs in hypertensive primary care attendees and should not be equated with diagnostic prevalence. Accordingly, our findings should not be interpreted as evidence that the CDT can reliably detect mild cognitive impairment or subtle early cognitive changes as a stand-alone instrument.

In the present study, although the CDT was administered by trained family physicians, its brief administration time and simple scoring suggest that it may be suitable for use in primary care settings following minimal training, rather than implying unsupervised routine use.

### Strengths of the study

One of the strengths of the study is the inclusion of a reasonably sized sample in the evaluation. The fact that the CDT was administered not as a single reference test but together with both the MMSE and the MoCA has provided a more accurate assessment of its diagnostic power. The separate evaluation of the 4-point and 0–1 versions of the CDT test, which have different scoring methods, has made it possible to comment on the prominent aspects of each scoring type in clinical practice.

### Limitations of the study

Outcome definition and reference standard. A key limitation of the present study is the absence of a clinical diagnostic reference standard for cognitive impairment. Participants were not evaluated using established clinical criteria (e.g., DSM-5 criteria for mild neurocognitive disorder, or consensus criteria for mild cognitive impairment), nor with a comprehensive neuropsychological assessment and clinical adjudication. Therefore, our findings should be interpreted as diagnostic accuracy of the CDT for identifying screen-positive cognitive impairment defined by predefined cut-offs on MoCA (primary) and MMSE (secondary), rather than validation against clinically diagnosed cognitive impairment. Although cut-offs derived from prior validation studies are widely used in primary-care research, they remain surrogate thresholds and may lead to misclassification (false positives/negatives), particularly across different educational backgrounds and cultural contexts. Future studies should evaluate CDT performance against a clinically adjudicated diagnosis and/or a standardized neuropsychological battery to better quantify its utility for case-finding in hypertensive populations.

Limitations of MMSE as a secondary reference threshold. We further acknowledge that MMSE < 24 while commonly applied as a screening threshold was originally established primarily to distinguish dementia from normal cognition and may have limited sensitivity for subtler or earlier cognitive deficits. Accordingly, we emphasize MoCA ≤ 21 as the primary reference screening threshold in this study, given its greater sensitivity to milder cognitive changes reported in the screening literature. Our MMSE-based analyses should therefore be viewed as complementary, reflecting lower global cognitive performance rather than an operational definition of early cognitive impairment. As these thresholds were derived from different case definitions (MMSE anchored in dementia–control contrasts vs. MoCA calibrated for milder impairment such as MCI), a higher screen-positive prevalence with MoCA than with MMSE is expected. Therefore, the present study was not designed to validate the CDT for clinically diagnosed mild cognitive impairment or for subtle early cognitive changes.

One of the significant limitations of our study is its cross-sectional design, which restricts our ability to draw causal inferences. The selection of cases from a single centre limits the generalisability of the results. Residual confounding by education cannot be fully excluded and may have influenced screening performance, despite our prespecified analytic approach. Because the study included only participants with at least primary school education, our findings cannot be generalized to illiterate individuals, and caution is warranted when interpreting CDT performance in elderly adults with low educational attainment.

In addition, the study was performed in a single-centre secondary-care outpatient setting with CDT administration by trained family physicians, which may restrict extrapolation to routine primary care practice. Future multi-centre studies embedded in real-world primary care workflows are needed to confirm diagnostic performance and implementation under typical time and staffing conditions.

## Conclusion

In this study, the CDT—particularly CDT-1 with a cut-off of ≤ 2—demonstrated high specificity with moderate sensitivity and concordance with established cognitive screening instruments when referenced against predefined MoCA and MMSE thresholds. These findings support the CDT as a practical and rapid pre-screening tool for identifying screen-positive cognitive impairment in hypertensive adults aged ≥ 50 years in primary care.

## Supplementary Information

Supplementary Material 1.

## Data Availability

The datasets generated and/or analysed during the current study are available in the Zenodo repository, https://doi.org/10.5281/zenodo.18014465.
