# Supplementary material for: Discriminative performance of the clock drawing test for screening cognitive function in hypertensive patients aged ≥ 50 years: a cross-sectional study
Source: BMC Prim Care. 2026 Apr 18;27:213. doi: 10.1186/s12875-026-03334-1 (PMC13220546; doi:10.1186/s12875-026-03334-1)
Supplement: Supplementary file 1 — Supplementary Material 1. [file 12875_2026_3334_MOESM1_ESM.docx]

**Supplementary Table 1. Comparison of participants and non-participants (decliners)**

| **Variable** | **Responding (n=365)** | **Non responding (n=77)** | **p** |
| --- | --- | --- | --- |
| **Age (mean±sd)** | 63.7±7.9 | 64.6±7.6 | 0.361^*^ |
| **Sex (Female n(%))** | 170 (46.5) | 35 (45.4) | 0.858^+^ |

*^* p value: Student’s t-test; + p value: chi-square test (Cramer’s V)^*
